# Supplementary figures and images for: The MIDAS domain of AAA mechanoenzyme Mdn1 forms catch bonds with two different substrates
Source: eLife. 2022 Feb 11;11:e73534. doi: 10.7554/eLife.73534 (PMC8837202; doi:10.7554/eLife.73534)

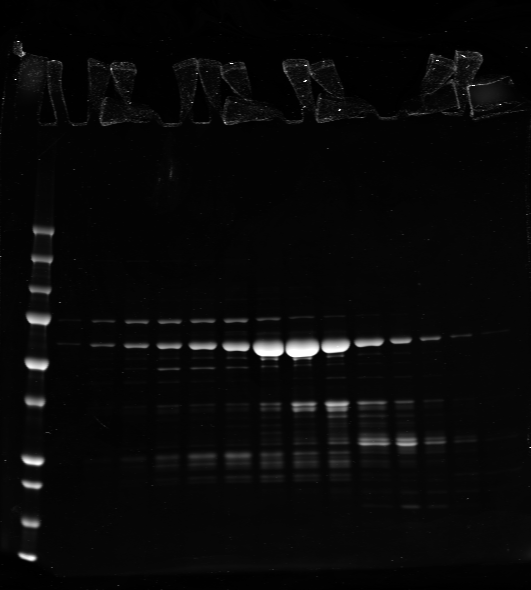

Supplement: Figure 1—source data 2. [file elife-73534-fig1-data2.zip › SourceData_Fig1_Gels/MIDAS-WT_Alone.TIF]

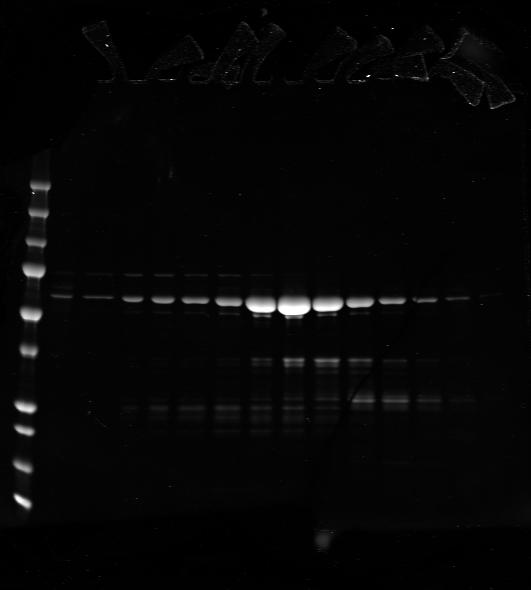

Supplement: Figure 1—source data 2. [file elife-73534-fig1-data2.zip › SourceData_Fig1_Gels/MIDAS-Y4666R-Alone.TIF]

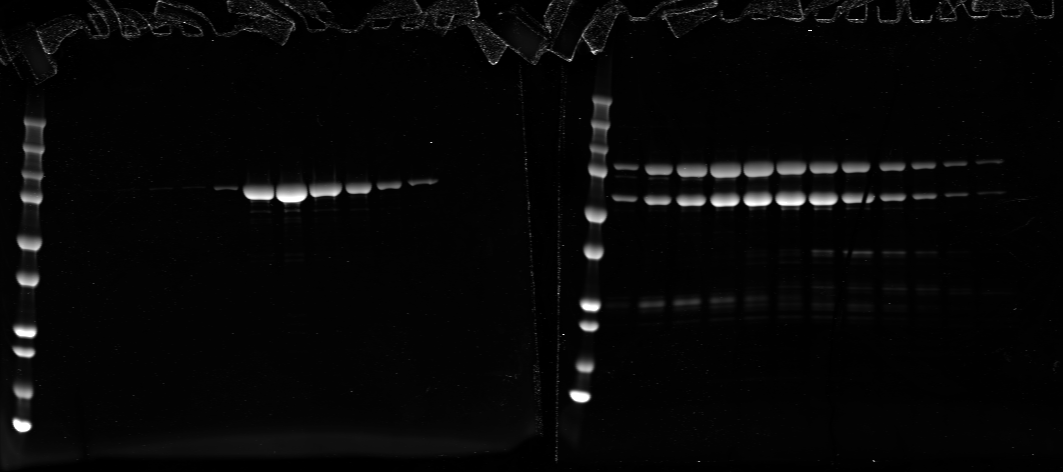

Supplement: Figure 1—source data 2. [file elife-73534-fig1-data2.zip › SourceData_Fig1_Gels/Rsa4-GFP Alone(left)_With MIDAS-WT(right).TIF]

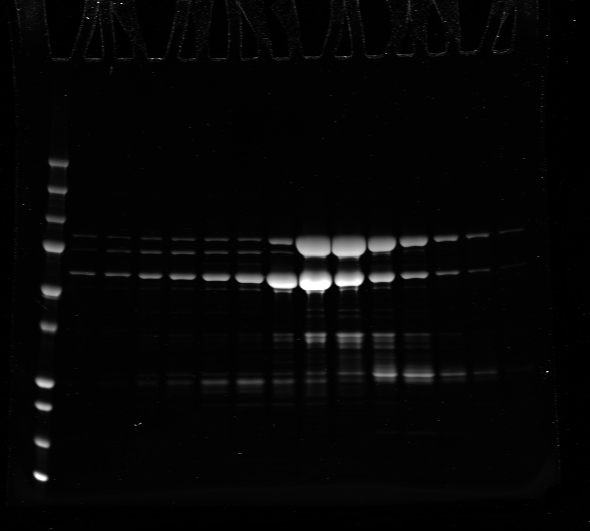

Supplement: Figure 1—source data 2. [file elife-73534-fig1-data2.zip › SourceData_Fig1_Gels/Rsa4-GFP with MIDAS-Y4666R.TIF]

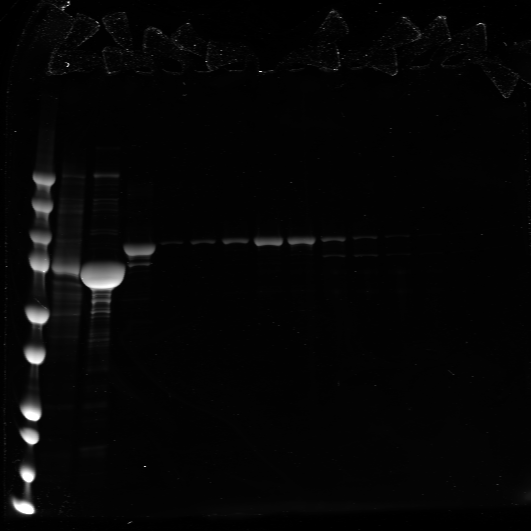

Supplement: Figure 3—source data 3. [file elife-73534-fig3-data3.zip › SourceData_Fig3S5_Gels/KJM_111621_Rsa4SNAP_PostOlgio_Final.TIF]

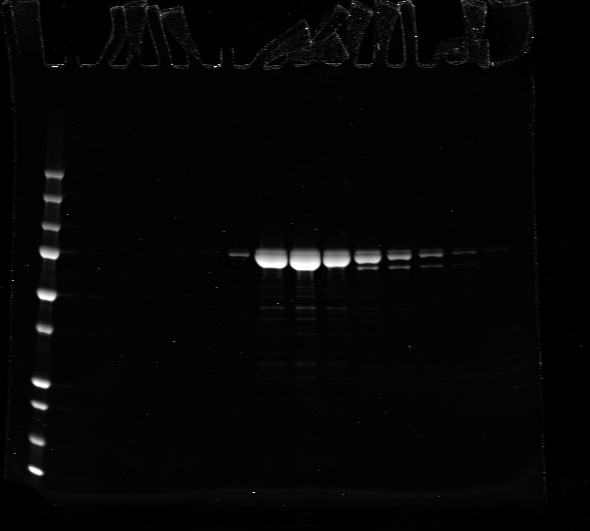

Supplement: Figure 4—source data 2. [file elife-73534-fig4-data2.zip › SourceData_Fig4_Gels/Ytm1-GFP alone.TIF]

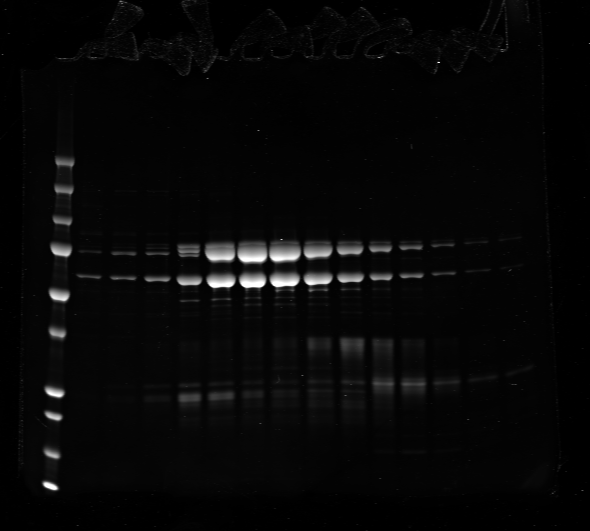

Supplement: Figure 4—source data 2. [file elife-73534-fig4-data2.zip › SourceData_Fig4_Gels/Ytm1-GFP with MIDAS-WT.TIF]

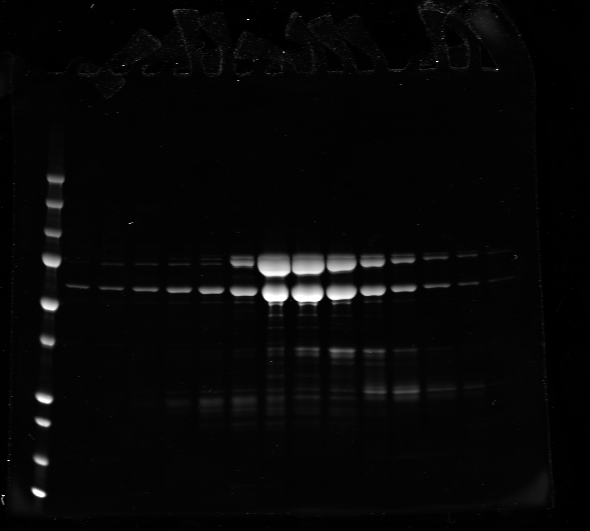

Supplement: Figure 4—source data 2. [file elife-73534-fig4-data2.zip › SourceData_Fig4_Gels/Ytm1-GFP with MIDAS-Y4666R.TIF]
